# Religious factors predict support for genomic medicine more strongly than politics, education, or trust: A survey of 4,939 adults in the United States

**DOI:** 10.3389/fgene.2025.1587774

**Published:** 2025-06-04

**Authors:** James M. DuBois, Eu Gene Chin, Erin D. Solomon, Jenine K. Harris, Peter Hill, Kari Baldwin, Lauren L. Baker

**Affiliations:** ^1^ Division of General Medicine and Geriatrics, Washington University in St. Louis, St. Louis, MO, United States; ^2^ Rosemead School of Psychology, Biola University, La Mirada, CA, United States; ^3^ Brown School, Washington University in St. Louis, St. Louis, MO, United States

**Keywords:** ELSI, genomics, survey research, ethics, religion & public life

## Abstract

**Background:**

Religious affiliation and attendance at services is associated with lower levels of support for some genomic activities, such as genetic testing. However, little is known about why or how religion shapes attitudes toward genomics.

**Materials and methods:**

We conducted a cross-sectional survey with 4,939 participants representative of nine religious groups in the US (including atheist and agnostic). The survey examined (a) attitudes toward diverse activities associated with genomic medicine, (b) religious beliefs and practices, (c) control variables including trust in the healthcare system and knowledge of genetics, and (d) demographics. We examined differences between groups using an Analysis of Covariance (ANCOVA), and developed a regression model to identify significant predictors of support for genetic medicine.

**Results:**

When controlling for demographic variables, only small attitudinal differences existed between religious groups, though substantial variability existed within groups. Only seven variables uniquely predicted attitudes toward genomic medicine: acceptance of evolution, support for promoting community health within their spiritual community, knowledge of genetics, more permissive attitudes toward reproduction and end of life care within their spiritual community, distrust in the healthcare system, political orientation, and frequency of volunteering (in descending order).

**Discussion:**

Our findings suggest that stereotyping based on religious affiliation is seriously misguided, and engagement with religious groups on genomic medicine must go beyond education and address moral issues and worldviews.

## Introduction

Religion is observed throughout all of human history and across cultures (Smith, 1981; Kim-Prieto, 2014). Despite declining rates of attendance at services (Pew Research Center, 2021), approximately 75% of people in the US still identify with a religion, and 45% say religion is very important in their lives (Gallup, 2024). Among those who are highly religious, their faith is the most important thing they consider when making major decisions or deciding what is right or wrong (Pew Research Center, 2018).

A growing consensus has developed that patient acceptance of genomics, novel vaccines, and public health measures will increase when healthcare professionals engage with patient’s perspectives (Wagner et al., 2016; Glasgow et al., 2018). Within the world of patient-centered outcomes research, “patient-centeredness is at its heart a question of including the worldview of patients” (Frank et al., 2014, p. 1513). From its inception, genomics has included investigation into its ethical, social, and legal implications (ELSI) (McEwen et al., 2014). The ELSI literature has grown significantly since that time, and has generate a body of empirical and policy literature that explores how adult and prenatal genetic testing is understood and used, and what unintended consequences it might have (Henneman et al., 2013; Jarvik et al., 2014; DuBois and Antes, 2015; Schneider et al., 2016; Cousens et al., 2017; Lazaro-Munoz et al., 2017; Sanderson et al., 2022; Iltis et al., 2023); whether genetic counseling is genuinely non-directive and how it is used by patients (Cadigan et al., 2011; Natoli et al., 2012; Jarvik et al., 2014; Stenehjem et al., 2018; Mayo-Gamble et al., 2019); ethical issues in gene editing (Allyse et al., 2015; Sankar and Cho, 2015; Michie and Allyse, 2019; Snure Beckman et al., 2019); and informed consent and privacy protections for biobanking and environmental data storage and use (Kaufman et al., 2008; Sanderson et al., 2017; Platt et al., 2018; Schwab et al., 2018; Lee et al., 2019; Staunton et al., 2019).

Nevertheless, the field of bioethics has been criticized for attempting to represent the interests of the public in health policy while losing touch with the actual views of the public, which are often deeply influenced by religious convictions (Evans, 2014).

Failure to engage patients and the public on matters of religion risks exacerbating health disparities through lower rates of engagement with genomic medicine and public health genomics. Specifically, it may increase disparities between religious and non-religious people but also disparities across races and the urban-rural divide: People belonging to several medically underserved communities—Black, Hispanic, and rural—are more likely to fall into the highly religious group which seeks to make major decisions informed by their faith (Pew Research Center, 2018; Association of Religion Data Archives, 2021).

Past studies indicate that religion is a strong predictor of concerns with genomic medicine and related technologies (Evans, 2006; Evans, 2014; Sayres et al., 2014; Allum et al., 2017; Scheufele et al., 2017; Critchley et al., 2019). However, these studies have generally focused only on religious affiliation and attendance at services; they do not indicate *why* religious affiliation is generally associated with lower support for genomic medicine. Moreover, most of these studies have focused on one technology or issue in genomic medicine—such as genetic testing—rather than the full array of activities associated with genomic medicine (Etchegary et al., 2010; Sayres et al., 2014; Allum et al., 2017; Scheufele et al., 2017; Critchley et al., 2019).

Because so little is known about why religion is associated with attitudes toward genomic medicine, we proposed a large sample exploratory study with two main research questions:

1. Are there significant differences between religious groups, including atheist and agnostic, regarding support for genomic medicine?
2. While taking into account demographic variables and other known predictors of attitudes, what specific features of religious or spiritual life uniquely predict support for genomic medicine?

Based on prior work in this area, in the present study we adopted a very broad concept of genomic medicine and public health genomics, one that includes six activities: (a) post-natal genetic testing, (b) storing and sharing biospecimens and health data, (c) genome editing, (d) stem cell therapy and research, (f) prenatal genetic testing, and (e) mRNA vaccines (Collins and Varmus, 2015; Jameson and Longo, 2015; Wagner et al., 2016; Sankar and Parker, 2017; Molster et al., 2018; DuBois et al., 2021). Together, these activities have contributed to significant health benefits, reducing infection rates, fostering innovative research, identifying health disorders, and more recently, even correcting genetic disorders (Manolio et al., 2024).

In selecting religious variables to examine, we drew upon several resources: (a) the project’s National Research Advisory Board, which is comprised of nine leaders working at the intersection of health and diverse religious traditions; (b) the guidance of a consultant who served as an editor of the encyclopedic work, *Measures of Religiosity and Spirituality* (Hill et al., 2026); and (c) a review of existing measures of variables of interest, including integration of faith with daily living, religious fundamentalism, views toward evolution and the theology of the body, religious discrimination, view toward God’s role in determining health, and Pew survey items on religious affiliation and practice (Hoge, 1972; Rutledge and Warden, 1999; Wallston et al., 1999; Altemeyer and Hunsberger, 2004; Mahoney et al., 2005; Pew Research Center, 2014; Kawika Allen et al., 2020).

## Materials and methods

### Study design

This study used a non-experimental, cross-sectional design, which is appropriate for exploratory studies aimed at testing differences between groups and building predictive models (Vogt et al., 2012). Participants completed a battery of measures in an online survey.

Because some of our analytical decisions were necessarily data-driven, we share some data in our Methods section, while reserving primary findings for the Results section.

### Data collection methods

The online survey battery consisted of measures assessing participants’ religious and spiritual beliefs and practices, which we have named the Spiritual Portrait Questionnaire. They also completed the Attitudes toward Genomics and Precision Medicine measure, measures of genetic knowledge, healthcare system distrust, and religious discrimination, and items pertaining to participant demographics. Further details of these measure are described in Supplementary Material.

### Sample characteristics

Participants were recruited using two panel service companies, Prolific and Cloud Research. From Prolific, we recruited participants who were representative of the US population in terms of age, gender, and race (*n* = 2,999). From Cloud Research, we recruited stratified samples of at least 300 participants from each of six religious groups: Black Protestant (e.g., African Methodist Episcopalian and National Baptist Convention), Catholic, Evangelical Protestant (e.g., Assemblies of God, Church of Christ, and Southern Baptist), Jewish (e.g., Conservative, Orthodox, and Reform), Mainline Protestant (e.g., Methodist, Episcopal, and Lutheran), and Muslim (e.g., Shi’a and Sunni) (*n* = 1,940) (Pew Research Center, 2025). To qualify for the study, participants needed to be 18 years or older and located in the United States. After removing participants who did not pass preliminary quality checks, our total sample size was *N* = 4,939 participants. Table 1 lists demographic details and composition of religious groups.

**TABLE 1:** Religious, political, and demographic characteristics (*N* = 4,939).

Sample characteristics	Mean	SD
Age in years (*n* = 7 or 0.14% missing)	46.4	17.0
Political orientation	4.4	2.4
	*n*	%
Gender		
Female	2,487	50.4
Male	2,403	48.7
Other	49	1.0
Race		
Native American	80	1.6
Asian/Asian American	275	5.6
Black	860	17.4
Pacific Islander	17	0.3
White	3672	74.4
^a^Ethnicity: Not Hispanic or Latino	4,589	92.9
Urban-rural home location		
Suburban	2,461	49.8
Urban	1,610	32.6
Rural	868	17.6
Employment		
Employed full time	2086	42.2
Employed part-time	683	13.8
Caregiver or homemaker	195	3.9
Self-employed	487	9.9
Retired	870	17.6
Unemployed	458	9.3
Other	160	3.2
Religious/non-religious group		
Catholic	807	16.3
Atheist	498	10.1
Agnostic	510	10.3
Jewish	381	7.7
Muslim	336	6.8
Spiritual	411	8.3
Evangelical protestant	536	10.9
Mainline protestant	541	11.0
Black protestant	419	8.5
Other	500	10.12
Political party		
Republican	1,126	22.8
Independent	1,357	27.5
Democrat	2,456	49.7
Education		
Less than High School	42	0.9
High School	662	13.4
Some College	1,053	21.3
Associates	525	10.6
Bachelors	1,696	34.3
Masters	730	14.8
Doctoral	202	4.1
Other	29	0.59
^a^Household Income > $50,000	2,898	58.7
Prefer not to answer	103	2.09

Political Orientation ranged from 1-“Extremely Liberal” through 9-“Extremely Conservative.” Age ranged from 18 to 88 years old.

^a^ See Online Only Supplementary Table S1 for additional categories.

### Survey administration

Participants completed the survey in Qualtrics, an online survey platform, in February, March, and April 2023. The survey took 30–45 min to complete. Within the survey, participants needed to correctly respond to two of three total attention check items presented (e.g., “If you are reading this item, please select the option strongly agree as your answer”).

### Ethical considerations

The study was approved (IRB #202201153) by the Institutional Review Board (IRB) at Washington University in St. Louis. Surveys were anonymous, using unique participant IDs provided by the survey panel companies. All data were stored in a password protected Box cloud storage folder.

### Statistical analysis

To answer the two main research questions, this study was divided into two major analytical sections. The first analytical section is comprised of an Analysis of Covariance (ANCOVA) model predicting general attitudinal support of genetic precision medicine. The second analytical section was comprised of a backward chunkwise elimination model building procedure (Kleinbaum et al., 2008) predicting general attitudinal support of genetic precision medicine. Supplementary Material provides further details on all variables that were included in the ANCOVA and backward chunkwise elimination model building procedure. Both analyses were conducted using IBM SPSS Statistics (Version 29).

Table 2 depicts the bivariate correlations between predictor variables used in the ANCOVA and backward chunkwise elimination model building procedures with overall attitudinal support for genomic medicine, which was calculated as the mean score of responses to the statements of general support for each of the six genomic medicine activities described in the AGPM. Scores thus ranged from 1 (strongly disagree) to 7 (strongly agree).

**TABLE 2:** Rank-ordered correlations of predictors with support for genomic medicine (*N* = 4,939).

Rank	Predictor variable	Overall support
1	Spiritual community’s permissive positions on reproductive and end of life views	0.39^*^
2	Spiritual community’s support for promoting community health	0.39^*^
3	Acceptance of evolution	0.37^*^
4	Conservative political orientation	−0.25^*^
5	Private prayer frequency	−0.20^*^
6	Religious fundamentalism	−0.20^*^
7	Distrust towards the healthcare system	−0.17^*^
8	Time spent in private prayer	−0.15^*^
9	Genetic knowledge index	−0.14^*^
10	Frequency of volunteering	0.11^*^
11	Age	−0.11^*^
12	Health in the last 4 weeks	−0.11^*^
13	Closet discrimination: Felt need to conceal religious identity from others	0.10^*^
14	Beliefs about God in the body	−0.09^*^
15	God and locus of control (belief that God controls everything)	−0.09^*^
16	Household income	0.09^*^
17	Education	0.08^*^
18	Integration of religious or spiritual beliefs in daily living	−0.07^*^
19	Attendance frequency in religious or spiritual group activities	0.03
20	Belief that others discriminate against them for their religion	−0.01
21	Frequency of practicing meditation	−0.01
22	Time spent in meditation	0.01

This table only includes scale and ordinal predictors that were used in the ANCOVA, and backward chunkwise elimination model building procedure.

*p < 0.001 (2-tailed).

#### Analysis 1: Analysis of covariance (ANCOVA)

The ANCOVA compared means of general attitudinal support for genetic precision medicine across the nine religious and non-religious groups while accounting for six demographic covariates. The religious and non-religious groups were Agnostic, Atheist, Black Protestant, Catholic, Evangelical Protestant, Jewish, Mainline Protestant, Muslim, and Spiritual but not religious. The demographic covariates were age in years, education, household income, urban/suburban/rural status, political orientation, and employment. The covariates were selected *a priori* based on the research team’s evaluation of previous related literature. Five hundred participants were excluded because they did not belong to one of the nine religious/non-religious groups—e.g., they reported being Orthodox Christian, no denominational affiliation, Mormon, Jehovah’s witness, Buddhist, Hindu—as these subgroup samples were too small to analyze. Consistent with safe harbor privacy rules, we excluded respondents who reported age ≥89 (*n* = 7). Additionally, 167 participants were excluded when responses could not be used in statistical analyses, such as “other,” “prefer not to answer,” or “more than one” on education, income, and race respectively. Because the exclusionary criteria were not mutually exclusive for the ANCOVA subsample (e.g., a participant may be age >89 and education listed as “other”), the total number of number of participants removed was only 623; and our total sample size for the ANCOVA was thus 4,316.

#### Analysis 2: Backward chunkwise elimination regression model building

The backward chunkwise elimination procedure was conducted to build a regression model for predicting attitudinal support for genetic precision medicine based on religious or spiritual variables, accounting for (1) demographic variables, (2) religious group, and (3) other general covariates known to predict attitudes in the literature. The research team identified 28 prospective predictor variables to include in the maximum model, which could be broadly divided into four categories (demographic variables, religious group, religious or spiritual predictors, or general covariates; Supplementary Material, pp. 5–10).

Because the model building procedure involved religious or spiritual variables, participants who reported being atheistic or agnostic (*n* = 1,008) were excluded from the model building procedure because they did not complete questionnaires related to religious or spiritual predictors. Additionally, participants who could not be categorized into any of the religious or non-religious categories (*n* = 500) were excluded from the model building procedures–leaving us with *N* = 3,431 participants. Subsequently, this subset of participants was randomly divided into a training group (*n* = 1,715) and holdout group (*n* = 1,716). Further details about model reliability indices can be found in Supplementary Material (p. 12). A backward chunkwise elimination model building procedure was conducted in the training group sample (Kleinbaum et al., 2008). More details about the backward chunkwise elimination procedure are described in the Supplementary Material (p. 13).

## Results

### Dispersion of mean support scores

**FIGURE 1:**
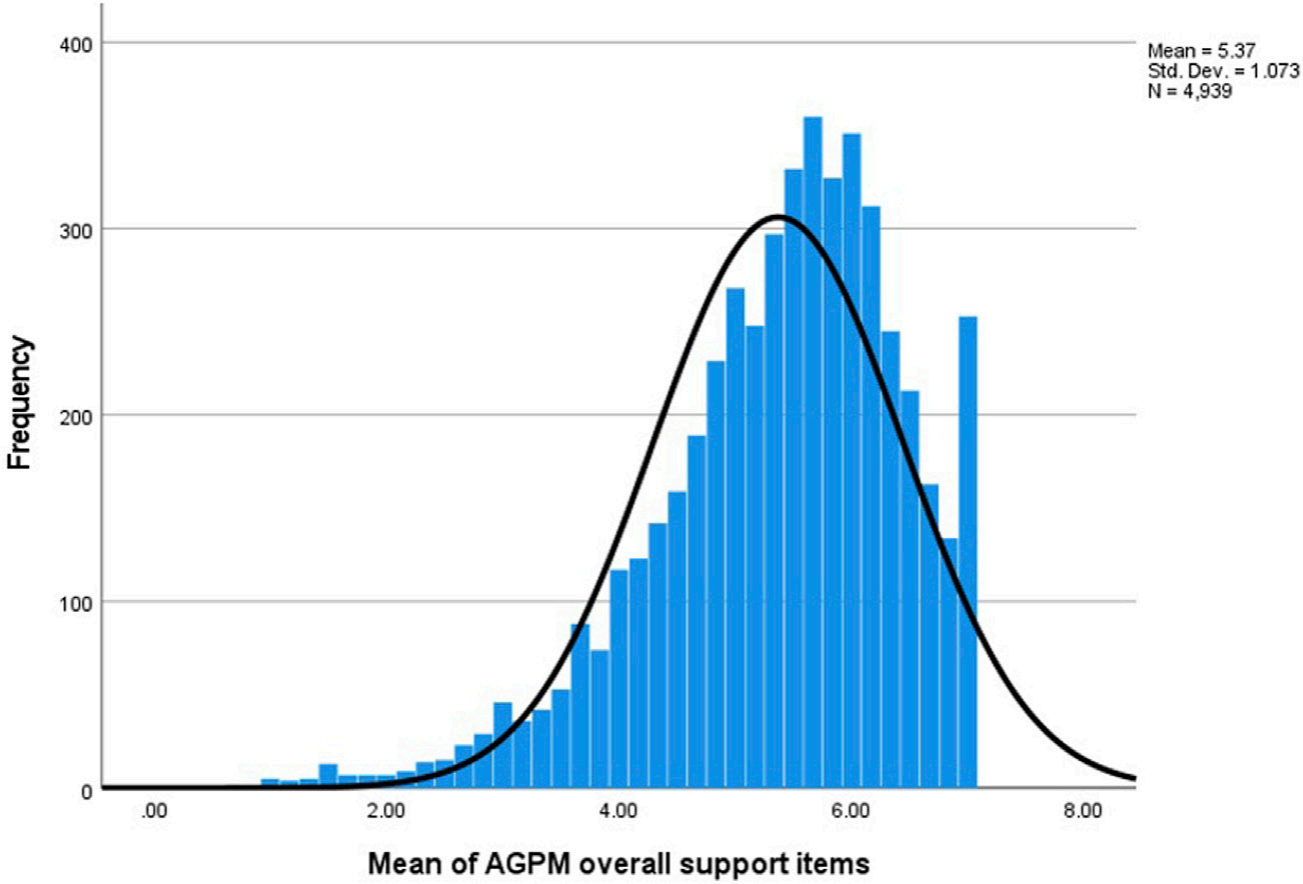
Dispersion of participants’ overall support (1–7) for genomic and medicine activities (Supplementary Figure S1 illustrates the distribution of “support” responses at the level of specific activities).

Figure 1 presents the distribution of participants’ mean level of support across all six genomic and precision medicine activities (1 strongly disagree – seven strongly agree) with higher scores indicating higher levels of support. The mean score of 5.37 roughly corresponds to a response of “somewhat agree” (a score of 5) to the statement “I generally support [the activity]”. Only 12% of respondents had mean ratings at or below neutral. Supplementary Figure S1 shows the distribution of support across all six genomic and precision medicine activities.

#### Analysis 1: Are there significant differences between religious groups, including atheist and agnostic, regarding support for genomic medicine?

Prior to building the ANCOVA model, we assessed collinearity among the predictors by conducting preliminary pairwise correlations and ANOVA tests between demographic variables (i.e., age, education, household income, employment, urban/suburban/rural status, and political orientation). The relationship between age and employment had a large effect size ( $\varepsilon^{2}$ = 0.384). (Cohen, 2013) Consequently, we excluded employment as a covariate but included age as a predictor in the ANCOVA model as a conservative approach to obviate multicollinearity concerns. Supplementary Material (p. 14) contains further description of the ANCOVA assumption checking results; after removing employment from the model, all assumptions were met.

The ANCOVA results suggested statistically significant, but very small mean differences (partial $\eta^{2}$ = 0.020) across the religious and non-religious groups in terms of attitudinal support towards genetic precision medicine when controlling for demographic covariates such as age, education, and political orientation (*F* (84,302) = 11.14, *p* < 0.001). Pairwise comparison between groups suggested that the Atheist group had a significantly higher mean for attitudinal supports for genetic medicine compared to all other groups ( ∆ *M* ranged from 0.31 to 0.50, with all *p*s < 0.001) , except for the Agnostic group ( ∆ *M* = 0.14, *p* = 0.66). Table 3 depicts the estimated marginal means, standard deviation and number of participants in each group.

**TABLE 3:** Estimated marginal means for attitudinal support among religious and non-religious groups in 4,316 survey participants in 2023.

Group	M	SD	*N*
Evangelical protestant	5.22	1.01	525
Black protestant	5.24	0.98	410
Spiritual	5.31	0.98	396
Mainline protestant	5.32	1.00	521
Catholic	5.36	0.98	787
Jewish	5.37	0.97	364
Muslim	5.40	1.00	329
Agnostic	5.58	1.00	498
Atheist	5.71	1.01	486
		Total	4,316

The estimated marginal mean for groups controls for covariates such as age, education, and political orientation. Without controlling for covariates, differences between groups were larger.

#### Analysis 2: What specific features of religious or spiritual life uniquely predict support for genomic medicine?

Prior to the backward chunkwise elimination procedure, a total of 28 predictors were considered for inclusion in the maximum model (Supplementary Material, pp. 5–10). Based on the training sample (*n* = 1,715), we conducted preliminary correlations and ANOVAs between each predictor and attitudinal support to exclude predictors that were (1) not statistically significant (*p* < 0.05) and (2) had a small effect size (*r* < 0.1; $\varepsilon^{2}$ < 0.01; *R* ^*2*^ < 0.02). (Cohen, 1992; Cohen, 2013) These preliminary analyses suggested that four predictors should be removed from the maximum model: (1) closet discrimination or the belief that others would discriminate against them for the participant’s religion, (2) private prayer time, (3) frequency of attendance at religious activities, and (4) race. Additionally, employment was removed because it was deemed to be too collinear with age (*p* < 0.001, $\varepsilon^{2}$ = 0.43). Thus, the maximum model contained 23 predictors grouped into 21 groups in order to (1) accommodate sets of dummy variables and (2) reduce the total number of predictors in the maximum model (Supplementary Material, p. 15).

Next, we sequentially removed 15 grouped predictors that produced a minimum test statistic *F*_*p*_ that was smaller than *F*_*CRIT*_ *,* where *α* = 0.002) (Šidák, 1967) until the minimum test statistic *F*_*p*_ in the model was larger than *F*_*CRIT*_, leaving us with six grouped predictors. The seven variables contained in these six groups were then subjected to a single variable backward elimination procedure using the same inclusion criterion (*α* = 0.002). All seven variables met the criterion for inclusion. Thus, these seven predictors were entered into a hierarchical regression (Table 4), arranged according to previously mentioned conceptual categories (see Supplementary Material pp. 5–10 for variable categories).

**TABLE 4:** Model for predicting support for the six genomic and precision medicine activities in a sample of *n* = 1,715.^a^

Model/predictor	*B*	*β*	*p*
General covariates			
Genetic knowledge	−0.16	−0.17	<0.001
Distrust towards the healthcare system	−0.19	−0.15	<0.001
Political orientation	−0.06	−0.12	<0.001
Religious variables			
Views on evolution	0.31	0.27	<0.001
Healthcare values of my spiritual community: reproductive and end of life issues	0.20	0.15	<0.001
Healthcare values of my spiritual community: community health	0.34	0.23	<0.001
Volunteer frequency	0.08	0.09	<0.001

	*R* ^***2***^	Δ*R* ^***2***^	
Step 1 general covariates Δ*F* (3, 1711) = 129.98	0.19	0.19	<0.001
Step 2 religious variables Δ*F* (4, 1707) = 130.92	0.38	0.19	<0.001
Overall *F* (7, 1707) = 147.44	0.38	-	<0.001

^a^ This model was then confirmed using a holdout sample of 1716.

Next, we evaluated the reliability of the model in the holdout sample (*n* = 1,716). We used the estimated prediction equation from the training sample (*n* = 1,715) to compute predicted values of the outcome in the holdout sample and determine the percentage relative shrinkage (Supplementary Material, p. 12). The shrinkage on cross-validation was 0.054, which was smaller than 0.10 suggested threshold (Kleinbaum et al., 2008), supporting the conclusion that this was a reliable model.

In summary, only four religious/spiritual variables remained as statistically significant predictors (*p* < 0.001) of support for genomic medicine after accounting for statistically significant general covariate variables, with a moderate effect size (Δ*R* ^*2*^ = 0.19) (Cohen, 2013) when all four variables were entered into the regression model (Table 4). Acceptance of evolution (*B* = 0.31, *p* < 0.001), permissive attitudes among their spiritual community regarding to reproductive (e.g., abortion) and end-of-life issues (e.g., euthanasia) (*B* = 0.20, *p* < 0.001), more favorable attitudes among their spiritual community regarding community health (*B* = 0.34, *p* < 0.001), and higher frequency of volunteer experience within their religious or spiritual group (e.g., help with food drives) (*B* = 0.08, *p* < 0.001) predicted stronger support for genetic precision medicine.

## Discussion

In a large sample that was nearly representative of the US in terms of age, gender, and race, with more than 300 participants representing each of the largest religious and non-religious groups in the US, we identified the variables that most strongly predicted support for genomic medicine. In descending order, these were: acceptance of evolution, support for promoting community health within their spiritual community, knowledge of genetics, and more permissive attitudes toward reproduction and end of life care within their spiritual community, distrust in the healthcare system, political orientation, and frequency of volunteering (Table 4). Many variables were examined but had no independent explanatory power such as level of education, income, age, gender, and race.

These findings are striking considering that the issues examined in this survey included elements of genomic healthcare that have been highly politicized (such as mRNA vaccines, stem cell research, and prenatal genetic testing). Despite being highly politicized, the influence of political orientation was weak when compared to several religious variables. Previous research has focused heavily on race, trust in the healthcare system, and level of education as possible predictors (Sayres et al., 2014; Critchley et al., 2019; Fisher et al., 2020); and indeed, we found that many of these variables were significantly correlated with attitudes toward genomic medicine (Table 2). However, by examining religious variables in more depth than is common and by using a rigorous regression design with a large sample, we found that these commonly examined factors generally paled in comparison with religiously-influenced variables. This aligns with the notion that faith is the most important consideration when making major decisions or deciding right or wrong among the highly religious (Pew Research Center, 2018).

At the same time, after controlling for covariates, the differences between religious and non-religious groups were very modest in terms of support for genomic medicine. That is, there were differences between religious groups, but they are not best explained in terms of group membership. In his post-mortem of the 2024 US Presidential election, David Brooks suggests that many people forecasted the results badly due to poor mental models. Specifically, he claims that the 2024 election results challenge a mental model in which “individual cognition is de-emphasized while collective consciousness is emphasized,” which views people “as members of a group before they are seen as individuals,” and where “groups are assumed to be relatively homogeneous” (Brooks, 2024). This study suggests that his intuition is correct: Most variation observed in this study was not across groups but within groups. The biggest difference in group mean support for genomic medicine that we observed—between atheists and Evangelical Protestants—was only 0.49, while the smallest standard deviation within a group was twice that size (0.97). This is an important finding: When it comes to attitudes toward genomic medicine, we must not make assumptions about people based on religious group membership.

How can this be reconciled with the finding that the strongest predictors of attitudes were religious—not political, educational, or racial? Specifically, how can this be reconciled with the fact that some of the strongest predictors of attitudes were precisely the values of their spiritual community? The groups that matter may be smaller, local, faith communities, which may not be homogenous within larger families of faith (e.g., Evangelical Protestant or Jewish). Further, within these groups there exist very different attitudes toward matters such as evolution and creation, and people may interpret their own traditions very differently.

On the matter of evolution, one might expect attitudes to be related to level of education. However, education level had no explanatory value in our models. Recent work by the sociologist, John Evans, may offer a key to understanding these findings. He suggests that the science-religion divide has less to do with methods of knowing the world and more to do with morality. His review of data indicates that “no religious group differs from the nonreligious comparison group in its propensity to seek out scientific knowledge,” but those who are religious may reject some claims that appear to contradict religious teachings, and above all, they have concerns about the moral agenda of researchers, or in our case, genomic medicine (e.g., regarding embryonic stem cell research, vaccine mandates, or prenatal genetic testing) (Evans, 2011, p. 707). It is possible that Evans’ findings also help explain how it is possible that religious fundamentalism and views about God and the body were not significant predictors of support for genomic medicine in our model: Fundamentalism and related beliefs do not diminish support unless they are accompanied by a rejection of scientific claims that appear to contradict religious beliefs. While scientists may not think fundamentalist beliefs and scientific beliefs can coexist, it appears that they can in fact (Evans, 2018).

Our study, combined with these insights from Evans, suggests that engaging the public with information only—that is educating the public on the nature and benefits of various genomic medicine activities—may be radically insufficient to increase acceptance of these activities within religious circles. Differences in how individuals evaluate these activities and technologies may rest more heavily on worldviews and morals than on factual knowledge. Effective engagement with religious communities—or individuals with religious convictions—may require two-way listening. Further, we may need to accept that some activities—despite their personal health or public health benefits—may never be acceptable to some segments of society.

As the United States and the international community observe rapid and radical changes in healthcare and health research policies with the start of President Trump’s second term, it is clear that we are in the midst of a so-called culture war (Messerly, 2025). But in healthcare, there is no room for cultural combat against patients or research participants. Peaceful and constructive engagement begins with understanding. We believe the current study greatly advances understanding by uncovering a lack of major attitudinal differences due to religious affiliation, and a diversity of views within groups. This is not to say that religious convictions do not matter–some religious convictions matter more than political and educational differences. Increasing awareness of these convictions and social dynamics within groups will be crucial to effective engagement with religious communities and individuals.

Limitations of the current study include the following: The sample was somewhat skewed toward more educated and more liberal individuals, which affects the generalizability of our descriptive statistics, though our sample size and statistical approach make it unlikely any inferential conclusions would change. Our sample sizes of specific racial and ethnic groups make it impossible to examine them as religious subgroups. Future research would benefit from stratified or purposive sampling by race and religion (Critchley et al., 2019). It might also examine how loyalty (or in-group vs. out-group thinking) moderates the effects of the values of one’s spiritual community regarding genomic medicine, which was a strong predictor in this study. Finally, our study’s measurement of certain predictor variables were brief when they were not the primary area of focus. For example, our measure of genetic knowledge focused on genetics, rather than, for example, the logistics of genetic testing and biobanking; our measures of political orientation included party affiliation and self-described liberal-conservative orientation, but did not assess group loyalty, which characterizes many people’s style of political engagement (Graham et al., 2009). Other variables, such as whether participants themselves or their families members have a genetic disorder, were not explored in this paper given the need to control the length of the survey, which required 30–45 min to complete.

In a follow up qualitative interview project, we are examining to what extent faith leaders (*N* = ∼160) from the six religious groups examined in this survey believe that religious teachings on prenatal genetic testing and vaccines are central and unchanging or rather might change with new information. We are also asking faith leaders what would constitute constructive and respectful public health engagement with their faith communities. Respecting the worldview of patients is important for reasons of ethics and effective engagement (Frank et al., 2014), yet one hopes that if beliefs are based on misinformation rather than core values or religious beliefs, then they might be subject to revision.

## Data Availability

The raw data supporting the conclusions of this article will be made available by the authors, without undue reservation.
