# Supplementary material for: Religious factors predict support for genomic medicine more strongly than politics, education, or trust: A survey of 4,939 adults in the United States
Source: Front Genet. 2025 Jun 4;16:1587774. doi: 10.3389/fgene.2025.1587774 (PMC12174436; doi:10.3389/fgene.2025.1587774)
Supplement: Supplementary file 1 [file Supplementaryfile1.docx]

**Table of Contents for Supplemental Material**

| Section | Page Number |
| --- | --- |
| Description of Measures | 2 |
| Supplemental Table 1. Predictor and Outcome Variables used in the Analysis of Covariance (ANCOVA) or Backward Chunkwise Elimination Regression Model | 5-10 |
| Supplemental Figure 1. Distribution of Attitudinal Support for Each of the Six Genomic Medicine Activities | 11 |
| Steps for Evaluating Reliability of Model from Backward Chunkwise Elimination Procedure | 12 |
| Backward Chunkwise Elimination Model Building Procedure | 13 |
| Assumption Checking for Analysis of Covariance (ANCOVA) Model | 14 |
| Supplemental Table 3. Maximum Model for Predicting Attitudinal Support | 15 |
| Assumption Checking for Regression Model Predicting Attitudinal Supports about Genetic Precision Medicine  Supplemental Figure 2. Breakdown of Samples | 16  17 |
| Supplement References | 18 |

**Description of Measures**

**Spiritual Portrait Battery of Questionnaires**

***Frequency of prayer and meditation.*** We used the item “During the average week of the past month, I prayed approximately…” and the analogous item for meditation to measure participants prayer and meditation practices. There were five response options, which ranged from 1=*0 or no days* to 5=*seven* *days*. Time spent praying and mediating were measures in minutes each day.

***Frequency attending religious services.*** We used the item: “How frequently do you attend religious or spiritual group services (e.g., go to events or services at a church, synagogue, mosque, temple, or other place of worship—excluding for weddings and funerals)?”

***Frequency of Volunteering***. We used the item: “How frequently do you volunteer to help within your religious or spiritual group (e.g., help with food drives, help with religious education activities, etc.)?” There were 5 response options ranging from 1=*never* to 5=*more than once per week*.

***Integration with daily living.*** We used 4 items from the Intrinsic Religious Motivation Scale to measure how integrated religion was to participant’s daily lives.^1^ Items were statements that participants rated on a 1-5 Likert-type scale (1=*strongly disagree,* 5=*strongly agree*). A sample item was “My religious or spiritual perspective shapes how I think and act each and every day.” We calculated a mean for each participant. Cronbach’s alpha was α=.90.

***Religious fundamentalism.*** We used 5 items from the Revised Religious Fundamentalism Scale to measure religious fundamentalism.^2^ Items were statements that participants rated on a 1-5 Likert-type scale (1=*strongly disagree,* 5=*strongly agree*). A sample item was “My religion provides a complete, unfailing guide to happiness and salvation, which must be totally followed.” We calculated a mean for each participant. Although the revised measure contained six items, internal consistency was very low (α=.58). Consequently, we removed item #2 “No single book of religious teachings contains all the intrinsic, fundamental truths about life” to improve the internal consistency of the measure (α=.86).

***Acceptance of evolution.*** We used 4 items from the Measure of Acceptance of the Theory of Evolution to measure participants views on human nature and creation.^3^ Items were statements that participants rated on a 1-5 Likert-type scale (1=*strongly disagree,* 5=*strongly agree*). A sample item was “Human beings are the product of evolutionary processes that have occurred over millions of years.” We calculated a mean for each participant, and higher scores indicate more acceptance of evolution. Cronbach’s alpha was α=.77.

***God in the body.*** We used 3 items from Mahoney et al. to measure participant’s views of their bodies as manifestations of God.^4^ Items were statements that participants rated on a 1-5 Likert-type scale (1=*strongly disagree,* 5=*strongly agree*). A sample item was “My body is created in God's image.” We calculated a mean for each participant. Cronbach’s alpha was α=.86.

***God and locus of control****.* We used 3 items from the God Locus of Health Control Scale to measure participant’s beliefs that God is the locus of control of one’s health.^5^ Items were statements that participants rated on a 1-6 Likert-type scale (1=*strongly disagree,* 6=*strongly agree*). A sample item was “Whatever happens to my health is God's will.” We calculated a mean for each participant. Cronbach’s alpha was α=.94.

***Healthcare values of my spiritual community.*** Our team developed a 13-item measure of Healthcare Values of My Spiritual Community (HVSC). The items were statements that participants rated on a 1-5 Likert-type scale (1=*strongly disagree,* 5=*strongly agree*). A sample item was “My religious or spiritual community supports letting people follow their own sense of what is morally right or wrong when making healthcare decisions.” We pilot tested the measure, and factor analysis yielded two factors which became the subscales: a 9-item subscale of **Permissive Positions on Reproductive and End of Life Issues** subscale, and a 4-item **Support for Promoting Health of the Community** subscale. We calculated a mean for each participant for each subscale. Cronbach’s alpha for each subscale were α=.88 and α=.82, respectively.

**Attitudes toward Genomics and Precision Medicine v.23.** The Attitudes toward Genomics and Precision Medicine (AGPM) is a 41-item measure that describes 6 precision medicine activities: genetic testing, storing and sharing health information and data, gene editing, stem cell therapy and research, prenatal genetic testing, and mRNA vaccines.^6,7^ All items were statements that participants rated on a 1-7 Likert-type scale (1=*strongly disagree*, 7=*strongly agree*). For each activity, participants first rate their level of agreement with the item “I generally support [this activity]”. The mean of these 6 items represents the Overall Support subscale. The remaining items are grouped into 7 subscales resulting from factor analysis: sacredness concerns, privacy concerns, gene editing concerns, mRNA concerns, social justice concerns, health benefits, and knowledge benefits. The mean of the items in the first five factors was calculated to generate a Concerns subscale score. A sample item was “I worry that people will be required to get mRNA vaccines.” Cronbach’s alpha for the subscales ranged from α=.79 to α=.88

**Genetic Knowledge Index.** We used 6 items total; 4 were from Furr and Kelly’s Genetic Knowledge Index, and 2 items from Fitzgerald-Butt et al.’s genetic knowledge measure.^8,9^ Items were true/false, and participants earned 1 point for each correct answer. Thus, scores could range from 0-6 points. A sample item was “Only mothers can pass on genetic disorders.” The Kuder-Richardson-20 reliability index was α=.60.

**Healthcare System Distrust.** We used 3 items from the Health Care System Distrust Scale. ^10^. Items were statements that participants rated on a 1-5 Likert-type scale (1=*strongly disagree*, 5=*strongly agree*). A sample item was “The health care system covers up its mistakes.” We calculated a mean for each participant. Although the original measure contained 4 items, internal consistency was very low (α=.563. Consequently, we removed item #3 “Patients get the same medical treatment from the health care system, no matter who they are” to improve the internal consistency of the measure (α=.73).

**Religious Discrimination.** We used 6 items from the Religious Discrimination Scale, which were categorized into two subscales: Closet Symptoms and Negative Labels.^11^ Closet Symptoms covers the belief that they would need to conceal their religious identity from others, and Negative Labels covers the belief that others would discriminate against them for their religion. Items consisted of statements that participants rated on a 1-5 Likert-type scale (1=*never/not applicable*, 5=*always*). We calculated a mean for each participant for each subscale. Cronbach’s alpha for each subscale were α=.79 and α=.87, respectively.

**Demographics.** We collected demographic information including age, gender, race, ethnicity, education, employment status, and household income. We also collected political orientation (“Overall, where would you place yourself, on the following scale?” 1=*extremely liberal*, 9=*extremely* *conservative*), information about their health over the past four weeks (“Overall, how would you rate your health during the past 4 weeks?” 1=*excellent,* 6=*very poor),* and whether they currently lived in an urban, suburban, or rural location*.*

**Supplemental Table 1. Predictor and Outcome Variables used in the Analysis of Covariance (ANCOVA) or Backward Chunkwise Elimination Regression Model**

| No. | Variable | Description | Variable Type | Variable  Category | Response Choices |
| --- | --- | --- | --- | --- | --- |
| 1 | Age^d^ | What is your age? | Numeric | Demographic | Open-Ended Response |
| 2 | Gender^d^ | What is your gender? | Categorical | Demographic | Female^b^, Male, Other |
| 3 | Race^d^ | Which racial categories do you identify with? | Categorical | Demographic | White^b^, Native American, Asian, Black, Pacific Islander |
| 4 | Ethnicity^d^ | What is your ethnicity? | Categorical | Demographic | Not Hispanic or Latino^b^, Hispanic or Latino, Prefer Not to Answer |
| 5 | Urban, suburban, or rural status^d^ | What best describes where you currently live? | Categorical | Demographic | Suburban^b^, Urban, Rural |
| 6 | Employment | Current Employment Status. | Categorical | Demographic | Employed Part-Time, Employed Full Time, Caregiver or Homemaker, Self-employed, Retired, Unemployed, Other |
| 7 | Household Income^d^ | What best represents your total household income? | Numeric | Demographic | 1. $0 – 25,000 2. $25,001 – 50,000 3. $50,001-75,000 4. $75,001 – 100,000 5. $100,001 – 150,000 6. $Greater than $150,000 |
| 8 | Education^d^ | What is your highest level of education? | Numeric | Demographic | 1. Less than High School 2. High School 3. Some College 4. Associates 5. Bachelors 6. Masters 7. Doctoral |
| 9 | Identification with religious or non-religious group^d^ | What is your present religion, if any? | Categorical | Religious Group | 1. Catholic^b^, Atheist, Agnostic, Jewish, Muslim, Spiritual, Evangelical Protestant, Mainline Protestant, Black Protestant |
| 10 | Political Orientation^d^ | Where would you place yourself on the following scale? | Numeric | General Covariate | 1. 1-“Extremely Liberal” through 9-“Extremely Conservative” |
| 11 | Health in the last four weeks^d^ | Overall, how would you rate your health during the past 4 weeks? | Numeric | General Covariate | 1. Excellent 2. Very Good 3. Good 4. Fair 5. Poor 6. Very Poor |
| 12 | Genetic Knowledge Index^d^ | Higher scores suggest more knowledge about genetics. Example item: “Only mothers can pass on genetic disorders.” | Numeric | General Covariate | 1. Sum of Scores range from 0 through 6 |
| 13 | Distrust towards the health care system^d^ | Higher scores suggest more distrust towards the healthcare system. Example item: “The health care system covers up its mistakes.” | Numeric | General Covariate | 1. Mean Scores range from 1 – “Strongly Disagree” through 5 through “Strongly Agree” |
| 14 | Private Prayer Frequency^d^ | During the average week of the past month, I prayed approximately: | Numeric | Religious or Spiritual Predictor | 1. 0 or No Days 2. One or Two Days 3. Three or Four Days 4. Five or Six Days 5. Seven Days |
| 15 | Private Prayer Time^a,d^ | On days when I prayed, my total time spent in prayer was approximately: | Numeric | Religious or Spiritual Predictor | 1. Less than 1 minute 2. A Few Minutes 3. 10 Minutes 4. 20 Minutes 5. 30 Minutes 6. An Hour 7. More than an Hour |
| 16 | Meditation Frequency^d^ | During the average week of the past month, I meditated approximately: | Numeric | Religious or Spiritual Predictor | 1. 0 or No Days 2. One or Two Days 3. Three or Four Days 4. Five or Six Days 5. Seven Days |
| 17 | Meditation Time^a,d^ | On days when I meditated, my total time spent in meditation was approximately: | Numeric | Religious or Spiritual Predictor | 1. Less than 1 minute 2. A Few Minutes 3. 10 Minutes 4. 20 Minutes 5. 30 Minutes 6. An Hour 7. More than an Hour |
| 18 | Attendance frequency in religious or spiritual group activities^c,d^ | How frequently do you attend religious or spiritual group services (e.g., go to events or services at a church, synagogue, mosque, temple, or other place of worship—excluding for weddings and funerals)? | Numeric | Religious or Spiritual Predictor | 1. Never 2. A Few Times a Year 3. Once or Twice a Month 4. Once a Week 5. More than Once a Week |
| 19 | Frequency Volunteer^c,d^ | How frequently do you volunteer to help within your religious or spiritual group (e.g., help with food drives, help with religious education activities, etc.): | Numeric | Religious or Spiritual Predictor | 1. Never 2. A Few Times a Year 3. Once or Twice a Month 4. Once a Week   More than Once a Week |
| 20 | Integration of religious or spiritual beliefs in daily living^c,d^ | Higher scores suggest higher integration. Example item: “My religious or spiritual beliefs are what lie behind my whole approach to life.” | Numeric | Religious or Spiritual Predictor | Mean Scores range from 1 – “Strongly Disagree” through 5 through “Strongly Agree” |
| 21 | Fundamentalist religious beliefs^c,d^ | Higher scores suggest stronger fundamentalist religious beliefs. Example item: “My religion provides a complete, unfailing guide to happiness and salvation, which must be totally followed. | Numeric | Religious or Spiritual Predictor | Mean Scores range from 1 – “Strongly Disagree” through 5 through “Strongly Agree” |
| 22 | Acceptance of Evolution^c,d^ | Higher scores suggest higher endorsement of evolutionary beliefs. Example item: “Evolutionary theory is the result of sound scientific research.” | Numeric | Religious or Spiritual Predictor | Mean Scores range from 1 – “Strongly Disagree” through 5 through “Strongly Agree” |
| 23 | Beliefs about God in the body^c,d^ | Higher scores suggest stronger belief that their body reflects God’s divine attributes. Example item: “My body is created in God’s image.” | Numeric | Religious or Spiritual Predictor | Mean Scores range from 1 – “Strongly Disagree” through 5 through “Strongly Agree” |
| 24 | Belief that they would need to conceal their religious identity from others^c,d^ | Higher scores suggest stronger belief that they need to conceal their religious identity from others. Example item: “I do not feel free to express who I am religiously.” | Numeric | Religious or Spiritual Predictor | Mean Scores range from 1 – “Never” through 6 – “Always |
| 25 | Belief that others would discriminate against them for the respondent’s religion.^c,d^ | Higher scores suggest stronger belief that others would discriminate against them. Example item: “I have heard people make unfriendly remarks about my religion.” | Numeric | Religious or Spiritual Predictor | Mean Scores range from 1 – “Never” through 6 – “Always |
| 26 | God and locus of control (belief that God controls everything).^c,d^ | Higher scores suggest stronger belief that God controls everything. Example item: “Whatever happens to my health is God’s will.” | Numeric | Religious or Spiritual Predictor | Mean Scores range from 1 – “Strongly Disagree” through 6 through “Strongly Agree” |
| 27 | Healthcare values of my spiritual community: Permissive pertaining to reproductive and end of life issues.^c,d^ | Higher scores suggest perceived favorable attitudes among their spiritual community pertaining to reproductive or life issues. Example item: “My religious of spiritual community support access to birth control pills to prevent pregnancy.” | Numeric | Religious or Spiritual Predictor | Mean Scores range from 1 – “Strongly Disagree” through 5 through “Strongly Agree” |
| 28 | Healthcare values of my spiritual community pertaining to community health.^c,d^ | Higher scores suggest perceived favorable attitudes among their spiritual community pertaining to community health. Example item: “My religious of spiritual community support recognizing healthcare as a basic human right.” | Numeric | Religious or Spiritual Predictor | Mean Scores range from 1 – “Strongly Disagree” through 5 through “Strongly Agree” |
| 29 | General support for genetic precision medicine. | Higher scores suggest greater support for genetic precision medicine. Example item: “I generally support gene editing.” | Numeric | Attitudinal Outcome | Mean Scores range from 1 – “Strongly Disagree” through 7 through “Strongly Agree” |

*Note.*

^a^Items that query for duration of time spent in meditation or prayer were skipped if participants rated “0 or no days” during the average week in the past month for engagement in prayer or meditation

^b^These categories were used as reference categories if entered in the backward chunkwise elimination regression model

^c^Participants who indicated that they were atheist or agnostic did not complete these items that assessed for spiritual/religious constructs

^d^Prospective variables that were considered for inclusion in the maximum model predicting attitudinal supports

**
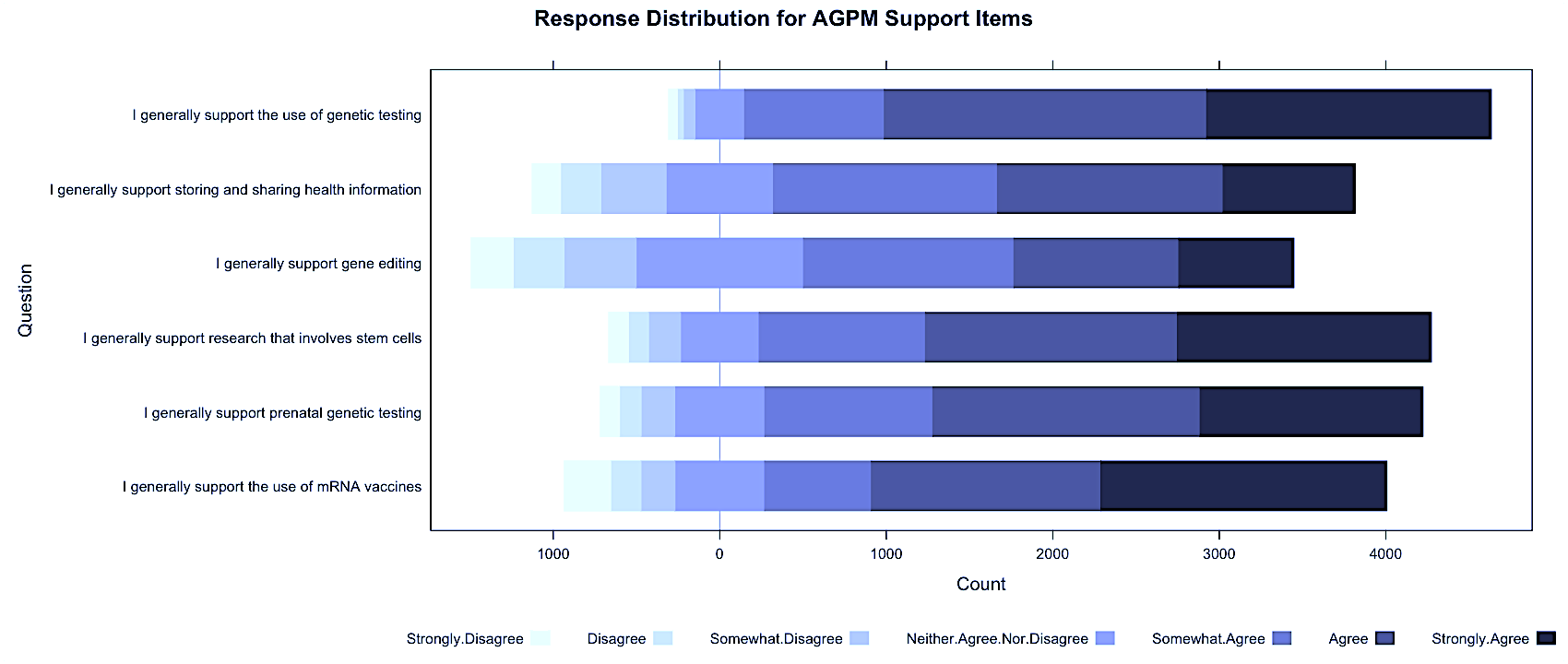
**

**Supplement Figure 1. Distribution of Attitudinal Support for Each of the Six Genomic Medicine Activities**

**Steps for Evaluating Reliability of Model from Backward Chunkwise Elimination Procedure**^12^

1. After conducting the backward chunkwise elimination procedure with the training dataset, we record the *R^2^* vector of parameter estimates from the finalized model.
2. Next, we compute $\hat{Y}$_2|1_ = X_2_$\hat{\beta}$_1_, where X_2_ is the holdout dataset. Essentially, we used the estimated prediction equation from the training group to compute predicted values of the outcome in the holdout sample.
3. Next, we compute the squared cross-validation correlation: $R_{*}^{2}$(2) *= r^2^*(*Y_2,_* $\hat{Y}$_2|1_)
4. Next, we compute the shrinkage on cross-validation: *R^2^*(1) - $R_{*}^{2}$(2)
5. While there are no firm rules as to how large a shrinkage must be to cast doubt on reliability, Kleinbaum et al.^12^ suggests that a fitted model is unreliable is the shrinkage is .90 or more, while values less than .10 indicate a reliable model.

**Backward Chunkwise Elimination Model Building Procedure^12^**

A backward chunkwise elimination model building procedure ^12^ was conducted to predict attitudinal supports in the training group sample. The elimination procedure was first applied to “chunks” of variables (i.e., variables grouped together for conceptual similarity), then applied to individual variables that remain in “chunks” that survived the initial round of elimination. For each round of elimination, the model building procedure consisted of five steps.

1. Specify the full model as the base, with *p* = *k* predictors, where *k* equals to the full number of chunks/variables in the model
2. Fit all *p*-1 variable models, defined by deleting one chunk/variable from the base model
3. For each model, compute the added-last test for the candidate chunk/variable
4. Find the minimum test statistics, *F_p._*
   - If statistically significant (i.e., *F_p_ > F_CRIT_*, where α is based on a Sidak-Bonferroni corrected test)^13^, stop and choose model *p*
   - If not statistically significant (i.e., *F_p_ < F_CRIT_*), delete the chunk/predictor with the minimum test statistic and reduce *p* by 1.
5. Go back to Step 2 and repeat the process

Once a final model was determined, we then evaluated the generalizability of the model by evaluating the previously mentioned shrinkage on cross validation value.

**Assumption Checking for Analysis of Covariance (ANCOVA) Model**

For the ANCOVA model predicting attitudinal supports for genetic precision medicine, examination of residual and normal probability plots did not suggest gross violations pertaining to linearity, normality, and homoscedasticity assumptions. Examination of interaction terms between each covariate and religious and non-religious groups suggested very small effect sizes (partial $\eta^{2}$s < .014)^19^, suggesting no gross violation pertaining to homogeneity of regression slopes for the ANCOVA.

**Supplemental Table 3. Maximum Model for Predicting Attitudinal Support**

| No. | Model/Predictor | *F* | *df* | *p* | Adjusted *R^2^*/$\Delta$*R^2^* |
| --- | --- | --- | --- | --- | --- |
|  | Maximum Model |  |  |  | .40 |
| 1 | Genetic Knowledge Index | 43.66 | 1.00 | <.001 | 0.02 |
| 2 | Distrust towards the health care system | 60.36 | 1.00 | <.001 | 0.02 |
| 3 | Integration of religious or spiritual beliefs in daily living | 1.03 | 1.00 | .31 | 0.00 |
| 4 | Religious fundamentalism | 12.43 | 1.00 | <.001 | 0.00 |
| 5 | Acceptance of evolution | 87.07 | 1.00 | <.001 | 0.03 |
| 6 | Beliefs about God in the body | 0.02 | 1.00 | .90 | 0.00 |
| 7 | Closet discrimination: Felt need to conceal religious identity from others | 0.15 | 1.00 | .70 | 0.00 |
| 8 | God and locus of control (belief that God controls everything) | 1.69 | 1.00 | .19 | 0.00 |
| 9 | Spiritual community’s permissive positions on reproductive & end of life views; and support for promoting community health. | 101.74 | 2.00 | <.001 | 0.07 |
| 10 | Private Prayer Frequency | 5.31 | 1.00 | .02 | 0.00 |
| 11 | Meditation Frequency; Meditation Time | 1.04 | 2.00 | .35 | 0.00 |
| 12 | Frequency Volunteer | 3.27 | 1.00 | .07 | 0.00 |
| 13 | Age | 2.73 | 1.00 | .10 | 0.00 |
| 14 | Health in the last four weeks | 1.46 | 1.00 | .23 | 0.00 |
| 15 | Education Level | 0.41 | 1.00 | .52 | 0.00 |
| 16 | Household Income | 19.75 | 1.00 | <.001 | 0.01 |
| 17 | Political Orientation | 32.64 | 1.00 | <.001 | 0.01 |
| 18 | Suburban^a^, urban, or rural status | 4.43 | 2.00 | .01 | 0.00 |
| 19 | Catholic^a^, Jewish, Muslim, Spiritual, EvangelicalProtestant, MainlineProtestant, BlackProtestant | 1.59 | 6.00 | .15 | 0.00 |
| 20 | Female^a^, male, other | 2.43 | 2.00 | .09 | 0.00 |
| 21 | Non-Hispanic/Latino^a^, HispanicLatino, PreferNoAnswer | 2.62 | 2.00 | .07 | 0.00 |

The full set of 23 predictors are grouped into 21 groups to accommodate dummy variables and reduce the overall number of predictors in the model.

^a^Reference categories

**Assumption Checking for Regression Models Predicting Attitudinal Supports Regarding Genetic Precision Medicine**

For the backward chunkwise elimination procedure predicting attitudinal supports, we only examined regression assumptions for the maximum model in the training sample and final model in the holdout sample.^12^ Examination of residual and normal probability plots for the maximum and final models did not suggest gross violations pertaining to linearity, normality, and homoscedasticity assumptions. Tolerance, variance inflation factor, and condition indices values suggested no significant multicollinearity concerns among the predictors in the maximum and final models.

**Supplemental Figure 2. Breakdown of Samples**

**Supplement References**

1. Hoge R. A validated intrinsic religious motivation scale. *Journal for the scientific study of religion*. 1972:369-376.

2. Altemeyer B, Hunsberger B. A Revised Religious Fundamentalism Scale: The Short and Sweet of It. *The International Journal for the Psychology of Religion*. 2004/01/01 2004;14(1):47-54. doi:10.1207/s15327582ijpr1401_4

3. Rutledge ML, Warden MA. The Development and Validation of the Measure of Acceptance of the Theory of Evolution Instrument. *School Science and Mathematics*. 1999;99(1):13-18. doi:<https://doi.org/10.1111/j.1949-8594.1999.tb17441.x>

4. Mahoney A, Carels RA, Pargament KI, et al. The Sanctification of the Body and Behavioral Health Patterns of College Students. *The International Journal for the Psychology of Religion*. 2005/07/01 2005;15(3):221-238. doi:10.1207/s15327582ijpr1503_3

5. Wallston KA, Malcarne VL, Flores L, et al. Does God Determine Your Health? The God Locus of Health Control Scale. *Cognitive Therapy and Research*. 1999;23(2):131-142.

6. DuBois JM, Mozersky J, Antes A, English T, Parsons MV, Baldwin K. Attitudes toward genomics and precision medicine. *J Clin Transl Sci*. 2021;5(1):e120. doi:10.1017/cts.2021.774

7. Harris JK, Solomon ED, Baldwin K, Baker LL, Chin EG, DuBois JM. [Pre-print] Integrating mRNA Vaccines into the Attitudes Toward Genomics and Precision Medicine Scale: A Validation Study with a Sample of 4,939 Adults in the US. Open Science Framework; 2024. doi:<https://doi.org/10.31219/osf.io/vczqb> August 20, 2024. Accessed November 12 2024. <https://doi.org/10.31219/osf.io/vczqb>

8. Furr LA, Kelly SE. The Genetic Knowledge Index: Developing a Standard Measure of Genetic Knowledge. *Genetic Testing*. 1999;3(2):193-199. doi:10.1089/gte.1999.3.193

9. Fitzgerald-Butt SM, Bodine A, Fry KM, et al. Measuring genetic knowledge: a brief survey instrument for adolescents and adults. *Clinical genetics*. 2016;89(2):235-243. doi:<https://doi.org/10.1111/cge.12618>

10. Shea JA, Micco E, Dean LT, McMurphy S, Schwartz JS, Armstrong K. Development of a revised Health Care System Distrust scale. *Journal of general internal medicine*. 2008;23(6):727-732. doi:10.1007/s11606-008-0575-3

11. Kawika Allen GE, Wang KT, Richards PS, Ming M, Suh HN. Religious Discrimination Scale: Development and Initial Psychometric Evaluation. *J Relig Health*. Apr 2020;59(2):700-713. doi:10.1007/s10943-018-0617-z

12. Kleinbaum DG, Kupper LL, Nizam A, Muller KE. *Applied Regression Analysis and Other Multivariable Methods*. 4th ed. Duxbury Press; 2008.

13. Šidák Z. Rectangular Confidence Regions for the Means of Multivariate Normal Distributions. *Journal of the American Statistical Association*. 1967;62(318):626-633. doi:10.1080/01621459.1967.10482935

14. *R: A Language and Environment for Statistical Computing*. R Foundation for Statistical Computing; 2023. <https://www.R-project.org/>

15. *dplyr: A Grammar of Data Manipulation*. Version 1.1.4. 2023. <https://CRAN.R-project.org/package=dplyr>

16. *BaylorEdPsych: R Package for Baylor University Educational Psychology*

*Quantitative Courses*. Version 0.5. 2012. <https://CRAN.R-project.org/package=BaylorEdPsych>

17. Little R, J. A. A Test of Missing Completely at Random for Multivariate Data with Missing Values. *Journal of the American Statistical Association*. 1988;83(404):1198-1202.

18. Bennett DA. How can I deal with missing data in my study? *Australian and New Zealand Journal of Public Health*. 2001;25(5):464-469. doi:10.1111/j.1467-842x.2001.tb00294.x

19. Cohen J. A power primer. *Psychological Bulletin*. 1992;112(1):155-159. doi:10.1037//0033-2909.112.1.155
